# Abnormal brain activities in multiple frequency bands in Parkinson’s disease with apathy

**DOI:** 10.3389/fnins.2022.975189

**Published:** 2022-10-10

**Authors:** Haikun Xu, Mengchao Zhang, Ziju Wang, Yanyan Yang, Ying Chang, Lin Liu

**Affiliations:** ^1^Department of Radiology, China-Japan Union Hospital of Jilin University, Changchun, China; ^2^Department of Pediatrics, China-Japan Union Hospital of Jilin University, Changchun, China; ^3^Department of Neurology, China-Japan Union Hospital of Jilin University, Changchun, China

**Keywords:** Parkinson’s disease, apathy, resting-state functional magnetic resonance, frequency specificity, intrinsic brain activity

## Abstract

**Background:**

Apathy is among the most prevalent and incapacitating non-motor symptoms of Parkinson’s disease (PD). PD patients with apathy (PD-A) have been reported to have abnormal spontaneous brain activity mainly in 0.01–0.08 Hz. However, the frequency-dependence of brain activity in PD-A remains unclear. Therefore, this study aimed to examine whether abnormalities in PD-A are associated with specific frequency bands.

**Materials and methods:**

Overall, 28 patients with PD-A, 19 PD patients without apathy (PD-NA), and 32 gender-, age-matched healthy controls (HCs) were enrolled. We collected resting-state functional magnetic resonance imaging (rs-fMRI) data, demographic information, and neuropsychological assessments, including apathy, depression, anxiety and cognitive function for every participant. The amplitude of low-frequency fluctuation (ALFF), fractional amplitude of low-frequency fluctuation (fALFF), percent amplitude of fluctuation (PerAF), regional homogeneity (ReHo), and degree centrality (DC) were calculated in the conventional (0.01–0.08 Hz), slow-4 (0.027–0.073 Hz), and slow-5 (0.01–0.027 Hz) frequency bands based on statistical parametric mapping (SPM12) and RESTplus V1.25. Two-sample *t*-tests were performed to compare the differences among the three groups.

**Results:**

PD-A reduced ALFF in the right anterior cingulate gyri in the slow-5 band and decreased fALFF in the right middle frontal gyrus in the conventional band, compared to patients with PD-NA. However, PerAF, ReHo, and DC could not distinguish PD-A from PD-NA in the three bands. PD-A had higher ALFF and fALFF in the left middle occipital gyrus and lower fALFF in the bilateral insula in the slow-5 band compared to the HCs. Furthermore, abnormal DC value in hippocampus and parahippocampus was observed separately in the conventional band and in the slow-4 band between PD-A and HCs. Moreover, PD-A and PD-NA showed lower ReHo in cerebellum in the three bands compared to the HCs.

**Conclusion:**

Our study revealed that PD-A and PD-NA might have different neurophysiological mechanisms. Concurrently, the ALFF in the slow-5 band and fALFF in the conventional band were sensitive in differentiating PD-A from PD-NA. The influence of apathy on the disease can be considered in the future research on PD, with the effects of frequency band taken into account when analyzing spontaneous brain activities in PD-A.

## Introduction

Apathy is defined as a state of diminished motivation, reducing interest for pleasurable activities and flattened affect (Marin, 1990; Kirsch-Darrow et al., 2006; Levy and Dubois, 2006). It is one of the most prevalent and disabling non-motor symptoms of Parkinson’s disease (PD) (Skorvanek et al., 2013; Pagonabarraga and Kulisevsky, 2017), which affects approximately 40% of PD patients (den Brok et al., 2015). Apathy in PD has been linked with a lower quality of life and increased caregiver burden (Pedersen et al., 2009). However, the pathophysiology of apathy in PD is still unclear. Therefore, a timely understanding apathy is critical to enable early intervention.

Resting-state functional magnetic resonance imaging (rs-fMRI) is a generally recognized non-invasive neuroimaging technique which is based on blood oxygen level-dependent (BOLD) signals (Fox and Raichle, 2007). The amplitude of low-frequency fluctuation (ALFF) is a reliable metric that can detect local spontaneous brain activity (Yang et al., 2007). In contrast, fractional amplitude of low-frequency fluctuation (fALFF) is the ratio of the power spectrum of the low frequency to that of the entire frequency range (Zuo et al., 2010). Notably, fALFF is more sensitive and specific in detecting spontaneous brain activity and can suppress non-specific signal components in fMRI (Zou et al., 2008). Percent amplitude of fluctuation (PerAF) is the percentage of BOLD fluctuations relative to the mean BOLD signal intensity for each time point and averaged across the entire time series (Jia et al., 2020). In addition, it has advantages related to test-retest reliability in both intra-and inter-scanners (Yang et al., 2021). Regional homogeneity (ReHo) is a credible metric that can measure local functional synchronization of spontaneous neuronal activities (Zang et al., 2004), while degree centrality (DC) reflects the density of intrinsic functional connectivity across the entire brain (Buckner et al., 2009).

The abovementioned metrics have been widely used to study neurodegenerative diseases, including PD. For example, Skidmore et al. (2013) applied ALFF to investigate abnormal spontaneous brain activity of patients being affected by PD with apathy (PD-A) and revealed that apathy in PD is linked with abnormalities in the left supplementary motor, right orbitofrontal, and middle frontal cortices. Sun et al. (2020a) reported dysfunction in the bilateral nucleus accumbens, dorsal anterior cingulate cortex (ACC), and the left dorsolateral prefrontal cortex. Furthermore, Sun et al. (2020b) discovered variations in the ReHo value in the ACC and right caudate. However, only ALFF and ReHo have been used to examine PD-A until recently; moreover, these studies were focused on the conventional frequency band (0.01–0.08 Hz). According to Buzsaki and Draguhn (2004), BOLD fMRI signals can be categorized into five different frequency bands as follows: slow-6 (0–0.01 Hz), slow-5 (0.01–0.027 Hz), slow-4 (0.027–0.073 Hz), slow-3 (0.073–0.198 Hz), and slow-2 (0.198–0.25 Hz). However, slow-6, slow-3, and slow-2 bands were excluded in our study due to their sensitivity to physiological noise and white matter, but slow-4 and slow-5 bands, which are mostly related to the brain gray matter, were appropriate for correlation analysis between functional processing and diseases (Salvador et al., 2008; Zuo et al., 2010).

In this study, we aimed to apply five different metrics, with the three frequency bands: conventional, slow-4, and slow-5, to investigate the characteristics of spontaneous brain activity with apathy in patients with PD. This result may enable us to identify the major brain hubs that contribute to apathy in patients with PD.

## Materials and methods

### Participants

In total, 79 participants including 32 healthy controls (HCs) and 47 patients with PD were recruited from November 2020 to January 2022. The clinical diagnosis of PD was conducted following the UK Parkinson’s Disease Society Brain Bank criteria (Hughes et al., 1992). According to the Edinburgh Handedness Inventory, all participants were right-handed (Oldfield, 1971). The exclusion criteria for the patients with PD were as follows: (1) diagnosis uncertain for PD or suspicion of Parkinsonism syndrome; (2) cognitive dysfunction (Mini-Mental State Examination [MMSE] score < 24) (Folstein et al., 1975); (3) brain impairment, such as brain trauma, infection, surgery, cerebral infarction, and cerebral hemorrhage; (4) drug abuse and alcohol abuse; and (5) contraindications for magnetic resonance imaging (MRI) scanning. 47 patients with PD were categorized into 28 PD-A patients and 19 PD-NA patients following the 14-item self-report Apathy Scale (AS). (Detailed grouping criteria were observed in the **Clinical and Neuropsychological Evaluation section**).

Additionally, the inclusion criteria for the HC group included no history of neurological disorder, psychiatric illness or any neurological disorder, and no contraindications for MRI scanning. The study protocol was approved by the Ethics Review Committee of the China-Japan Union Hospital of Jilin University and was performed following the Declaration of Helsinki.

### Clinical and neuropsychological evaluation

Sex, age, and other demographic characteristics were obtained from all participants. Additionally, the participants were assessed using the MMSE, Hamilton Anxiety Scale (HAMA), Hamilton Depression Scale (HAMD), and AS. The MMSE was adopted to assess cognitive function. The HAMA and HAMD were used to evaluate individual’s anxious and depressive states. For PD patients, a comprehensive clinical assessment (including motor and non-motor symptoms) was conducted on all patients during the off-period (12 h after the patients stopped taking medication for PD). The severity of motor symptoms for each patient was evaluated using the Unified Parkinson’s Disease Rating Scale (UPDRS-III) (Goetz et al., 2007), and the disease stage was assessed based on the Hoehn and Yahr (H&Y) stage (Hoehn and Yahr, 1967). Apathy was evaluated with the 14-item self-report AS (Starkstein et al., 1992), which is a standard measure of apathy in PD (Leentjens et al., 2008). The AS scores range from 0 to 42, which categorizes patients with PD into apathy (AS score ≥ 14, PD-A) and non-apathy (AS score < 14, PD-NA) groups (Leentjens et al., 2008). The levodopa equivalent daily dose (LEDD) was estimated from PD patients.

### Imaging data acquisition

All MRI data were obtained on a 3 T MRI scanner (Skyra, Siemens Healthcare, Erlangen, Germany) with a 16-channel head coil. All patients with PD were scanned during the off-period (12 h after the patients stopped taking medication for PD). Head movement was reduced by foam padding, and earplugs were used to diminish the scanner noise. Additionally, the participants were required to rest with their eyes closed, focus on nothing in particular, and not fall asleep during the entire scanning process. Functional images were acquired by a simultaneous multi-slice (SMS) echo-planar imaging (EPI) sequence with the parameters as follows: repetition time (TR) = 1,500 ms, echo time (TE) = 30 ms, flip angle (FA) = 70°, matrix size = 112 × 112, slice thickness/gap = 2 mm/0.4 mm, slice acceleration factor = 4, parallel acceleration factor = 2, voxel size = 2 × 2 × 2 mm, field of view (FOV) = 224 mm × 224 mm, and slice numbers = 68. Overall, 340 brain volumes were obtained in this study. Furthermore, after the functional scan, high-resolution anatomical images were acquired sagittally using a 3D-magnetization-prepared rapid gradient-echo (MPRAGE) sequence with the following parameters: TR = 2,300 ms, TE = 2.98 ms, flip angle (FA) = 9°, matrix size = 248 × 256, slice thickness = 1 mm, no slice gap, voxel size = 1 × 1 × 1 mm, field of view (FOV) = 25 mm × 248 mm, and slice number = 176.

### Imaging data preprocessing

The rs-fMRI data were preprocessed using RESTplus 1.25 (Jia et al., 2019) based on statistical parametric mapping (SPM 12^1^), which was run on Matlab 2017b (MathWorks, Natick, MA, USA). The main steps included (1) excluding the first 10 volumes from the 340 volumes for stabilization; (2) slice timing correction; (3) head motion correction; (4) spatial normalization and resampling to 3 × 3 × 3 mm voxels; (5) spatial smoothing with an isotropic Gaussian kernel with a full width at half maximum (FWHM) of 6 mm (ReHo and DC values were finally smoothed); (6) removing the linear trend of the time course; and (7) nuisance covariate regression, which includes Friston-24 head motion parameters (Friston et al., 1996), white matter signal, and cerebrospinal fluid signal.

### Metrics calculation

We calculated the ALFF, fALFF, PerAF, ReHo, and DC within the conventional (0.01–0.08 Hz), slow-4 (0.027–0.073 Hz), and slow-5 (0.01–0.027 Hz) frequency bands, which was performed using the toolkits of RESTplus, version 1.25.

#### Voxel level metrics

Amplitude of low-frequency fluctuation (ALFF), fALFF, and PerAF were obtained with the following procedures: The time sequences were converted to the frequency domain using a fast Fourier transform. Subsequently, the square root of the power spectrum was calculated and averaged across each predefined frequency band, and the averaged square root in the voxel was considered as the ALFF (Zang et al., 2007). fALFF was derived by computing the power ratio of each predefined frequency range to that of the entire frequency band (Zou et al., 2008). PerAF was acquired by measuring the percentage of BOLD signal strengths relative to the average BOLD signal intensity at each time point and determining the average value for the entire time series (Jia et al., 2020).

#### Regional level metric

Regional homogeneity (ReHo) was calculated by employing Kendall’s coefficient of concordance to measure the similarity of the time series of a given voxel to its nearest 26 neighbors (Zang et al., 2004). Subsequently, the ReHo map was spatially smoothed with a 6 mm FWHM Gaussian kernel. Additionally, the ReHo value of each voxel was divided by the global mean ReHo for standardization, as mentioned above.

#### Whole-brain level metric

The binary DC was defined as the number of significant connections between a voxel’s time course and that of other voxels. We limited the analysis to positive correlations above a threshold of *r* = 0.25 (Buckner et al., 2009) to remove voxels with weak temporal correlations due to noise or white matter. Finally, the binary DC map was spatially smoothed with a 6 mm FWHM Gaussian kernel.

### Statistical analysis

Demographic data was analyzed using the Statistical Package for Social Sciences (SPSS 26.0). Chi-squared (χ2) and rank-sum tests were performed for categorical data. Kruskal–Wallis H and Mann–Whitney *U* tests were used to compare the non-normally distributed parameters, and normally distributed continuous variables were compared using one-way analysis of variance (ANOVA).

Statistical analyses of functional images were performed with SPM12 and DPABI 6.0 (Yan et al., 2016). For each frequency band, a two-sample *t*-test was performed to compare the ALFF, fALFF, PerAF, ReHo, and DC values for the PD-A vs. HCs, PD-NA vs. HCs, and PD-A vs. PD-NA groups within the gray matter mask. We restricted our analyses to a predefined gray matter mask with gray matter tissue probability greater than 50%, which was released as part of tissue priors in SPM12. The resultant T-maps were adjusted for multiple comparisons using the Gaussian random field (GRF) theory (voxel *P* < 0.01, cluster *P* < 0.05, two-tailed).

## Results

### Clinical and neuropsychological evaluation

There was no noted inter-group significant difference in sex (χ^2^ = 2.4, *P* = 0.30), age (*H* = 4.2, *P* = 0.12), and education (*F* = 1.0, *P* = 0.37). The MMSE (*H* = 19.0, *P* < 0.001), AS (*H* = 66.4, *P* < 0.001), HAMA (*H* = 45.9, *P* < 0.001), HAMD (*H* = 56.2, *P* < 0.001) varied significantly across the three groups. The inter-group difference for MMSE (*P* = 0.82), HAMA (*P* = 0.29), HAMD (*P* = 0.24) scores was not significantly different between PD-A and PD-NA groups. The MMSE (*P* < 0.001), HAMA (*P* < 0.001), HAMD (*P* < 0.001) scores were significantly different between PD-A and HC. Among PD-NA and HC groups, the MMSE (*P* = 0.09) were not significantly different, while the HAMA (*P* = 0.03), HMAD (*P* = 0.02) scores were different. The AS scores (*P* < 0.001) were significantly different among three groups. The duration of disease (*U* = −2.0, *P* = 0.04), the UPDRS-III scores (*U* = −2.5, *P* = 0.01), and the LEDD (*U* = −2.5, *P* = 0.01) were different between PD-A and PD-NA. Whereas no significant difference existed in the H&Y stage (χ^2^ = 5.3, *P* = 0.39) between the PD-A and PD-NA (Table 1).

**TABLE 1:** Demographic and neuropsychological information.

	PD-A	PD-NA	HCs	Static	*P*
Participants	28	19	32		
Male/female	10/18	11/8	13/19	2.4^a^	0.30
Age	65.1 (6.2)	60.3 (11.3)	63.2 (4.7)	4.2^b^	0.12
Education	9.9 (3.2)	11.2 (4.8)	9.8 (3.8)	1.0^c^	0.37
MMSE	26.6 (1.9)	27.1 (1.9)	28.7 (1.3)	19.0^b^	< 0.001^1,2,3^
AS	21.2 (4.7)	7.9 (2.9)	1.5 (2.5)	66.4^b^	<0.001^1,2,3^
HAMA	16.2 (8.2)	11.4 (10.9)	1.4 (2.1)	45.9^b^	< 0.001^1,2,3^
HAMD	20.1 (11.8)	13.4 (13.5)	0.9 (1.4)	56.2^b^	< 0.001^1,2,3^
Duration of disease	5.4 (4.5)	3.2 (3.3)	–	−2.0^d^	0.04
UPDRS-III	39.0 (18.2)	26.9 (13.5)	–	−2.5^d^	0.01
LEDD	584.1 (322.8)	322.4 (376.3)	–	−2.5^d^	0.01
H&Y stage	2.1 (0.8)	1.4 (0.5)	–	5.3^a^	0.39

The data are shown as the mean (standard deviation). PD-A, Parkinson’s disease with apathy; PD-NA, Parkinson’s disease without apathy; HCs, healthy controls; MMSE, Mini-Mental State Examination; AS, Apathy Scale; HAMA, Hamilton Anxiety Scale; HAMD, Hamilton Depression Scale; UPDRS-III, Part III of the Movement Disorder Society-sponsored revision of the Unified Parkinson’s Disease Rating Scale; LEDD, levodopa equivalent daily dose; H&Y stage, Hoehn and Yahr stage; a: Pearson’s chi-square (χ^2^); b: Kruskal–Wallis *H* test; c: One-way analysis of variance; d: Mann–Whitney *U* test; 1: *Post hoc* paired comparisons between PD-A and PD-NA groups; 2: *Post hoc* paired comparisons between PD-A and HC groups; 3: *Post hoc* paired comparisons between PD-NA and HC groups.

### Group differences in amplitude of low-frequency fluctuation map

**FIGURE 1:**
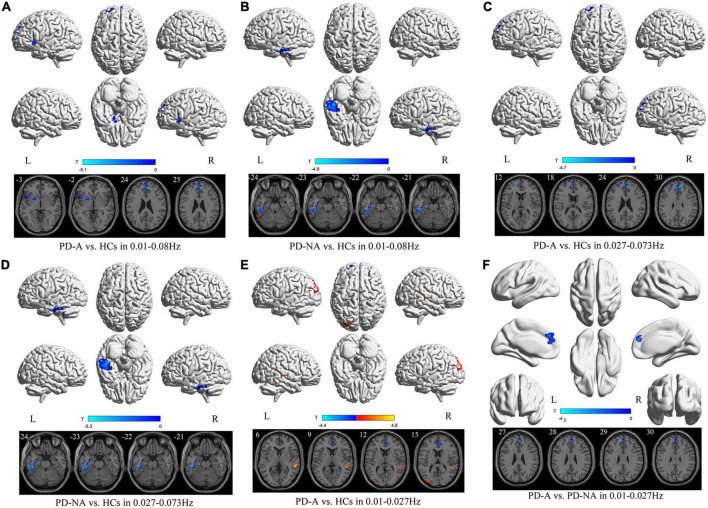
Amplitude of low-frequency fluctuation (ALFF) maps differences in three frequency bands among the groups (PD-A vs. HCs, PD-A vs. PD-NA, and PD-NA vs. HCs). The color bar on the bottom indicates the statistical *t* value. The warm (cold) color indicates a significantly increased (decreased) ALFF in the comparison.

In the conventional frequency band, the ALFF value in the left insula (*t* = −4.1) and right anterior cingulate gyri (*t* = −5.1) was decreased in patients with PD-A compared to HCs (Figure 1 and Table 2). However, no regions exhibited significant differences between the PD-A and PD-NA groups. The ALFF value was lower in the left inferior temporal gyrus (*t* = −4.9) in patients with PD-NA than in the HCs (Figure 1 and Table 2).

**TABLE 2:** Differences of amplitude of low-frequency fluctuation (ALFF) values in three frequency bands among the groups.

Contrast	Brain regions	BA	Volume (mm^3^)	MNI coordinates			*t*-value
				X	Y	Z	
**1. Conventional frequency band**							
PD-A vs. HCs	PD-A < HCs						
	left insula	48	2619	−39	15	−3	−4.1
	right anterior cingulate gyri	32	7155	6	45	24	−5.1
PD-A vs. PD-NA	NA						
PD-NA vs. HCs	PD-NA < HCs						
	left inferior temporal gyrus	20	4158	−42	−30	−24	−4.9
**2. Slow-4 band**							
PD-A vs. HCs	PD-A < HCs						
	right anterior cingulate gyri	32	3726	6	45	24	−4.7
	left superior frontal gyrus	10	2079	−21	57	12	−4.7
PD-A vs. PD-NA	NA						
PD-NA vs. HCs	PD-NA < HCs						
	left inferior temporal gyrus	20	4428	−42	−30	−24	−5.3
**3. Slow-5 band**							
PD-A vs. HCs	PD-A > HCs						
	right middle temporal gyrus	22	3132	57	−33	6	4.8
	left middle occipital gyrus	19	2835	−36	−81	15	4.2
	PD-A < HCs						
	left anterior cingulate gyri	32	5022	−9	39	12	−4.4
PD-A vs. PD-NA	PD-A < PD-NA						
	right anterior cingulate gyri	32	1701	3	45	27	−4.0
PD-NA vs. HCs	NA						

BA, Brodmann’s area; MNI, Montreal Neurological Institute; PD-A, Parkinson’s disease with apathy; PD-NA, Parkinson’s disease without apathy; HCs, healthy controls; NA, Not applicable; - no significant clusters detected.

In the slow-4 frequency band, there was decreased ALFF in the right anterior cingulate gyri (*t* = −4.7) and left superior frontal gyrus (*t* = −4.7) in patients with PD-A, relative to the HCs (Figure 1 and Table 2). No difference was observed between the PD-A and PD-NA groups. The ALFF value was decreased in the left inferior temporal gyrus (*t* = −5.3) in patients with PD-NA compared to HCs (Figure 1 and Table 2).

Furthermore, in the slow-5 frequency band, the patients with PD-A exhibited significantly higher ALFF in the right middle temporal gyrus (*t* = 4.8), left middle occipital gyrus (*t* = 4.2), and lower ALFF in the left anterior cingulate gyri (*t* = −4.4) than in the HCs (Figure 1 and Table 2). Patients with PD-A showed lower ALFF in the right anterior cingulate gyri (*t* = −4.0) than those with PD-NA (Figure 1 and Table 2). However, no difference was observed between the PD-NA and HCs.

### Group differences in fractional amplitude of low-frequency fluctuation map

**FIGURE 2:**
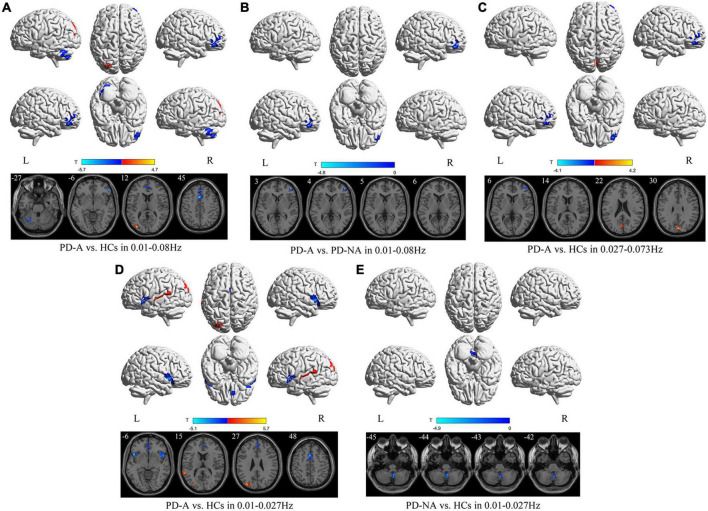
Fractional amplitude of low-frequency fluctuation (fALFF) maps differences among the groups (PD-A vs. HCs, PD-A vs. PD-NA, and PD-NA vs. HCs) in three frequency bands. The color bar on the bottom indicates the statistical *t* value. The warm (cold) color indicates a significantly increased (decreased) fALFF in the comparison.

In the conventional frequency band, there was decreased fALFF in the left cerebellum_6 (*t* = −4.4), right inferior frontal gyrus, orbital part (*t* = −4.9), right superior frontal gyrus, medial (*t* = −5.0), and left supplementary motor area (*t* = −5.7), along with increased fALFF in the left middle occipital gyrus (*t* = 4.7) in patients with PD-A compared to HCs (Figure 2 and Table 3). The fALFF value was decreased in the right middle frontal gyrus (*t* = −4.8) in patients with PD-A compared to those with PD-NA (Figure 2 and Table 3). However, no regions exhibited significant differences between the PD-NA and HCs.

**TABLE 3:** Differences of fractional amplitude of low-frequency fluctuation (fALFF) values in three frequency bands among the groups.

Contrast	Brain regions	BA	Volume (mm^3^)	MNI coordinates			*t*-value
				X	Y	Z	
**1. Conventional frequency band**							
PD-A vs. HCs	PD-A < HCs						
	left Cerebelum_6	37	1755	−30	−60	−27	−4.4
	right inferior frontal gyrus, orbital part	47	2133	51	36	−6	−4.9
	right superior frontal gyrus, medial	8	2457	3	36	42	−5.0
	left supplementary motor area	32	2457	−3	15	45	−5.7
	PD-A > HCs						
	left middle occipital gyrus	18	3483	−33	−84	12	4.7
PD-A vs. PD-NA	PD-A < PD-NA						
	right middle frontal gyrus	46	1296	45	48	3	−4.8
PD-NA vs. HCs	NA						
**2. Slow-4 band**							
	PD-A < HCs						
	right middle frontal gyrus	46	1512	45	54	6	−4.1
	PD-A > HCs						
	left cuneus	18	1458	0	−81	30	4.2
PD-A vs. PD-NA	NA						
PD-NA vs. HCs	NA						
**3. Slow-5 band**							
PD-A vs. HCs	PD-A < HCs						
	right insula		2754	51	12	−6	−4.7
	left insula	48	2349	−45	12	−3	−5.1
	left anterior cingulate gyri	10	1647	0	42	−3	−3.4
	left superior frontal gyrus, medial	32	2106	0	45	30	−4.1
	left supplementary motor area	32	2052	−3	12	48	−5.0
	PD-A > HCs						
	left superior temporal gyrus	22	1701	−60	−45	15	4.5
	left middle occipital gyrus	19	3618	−33	−81	27	5.7
PD-A vs. PD-NA	NA						
PD-NA vs. HCs	PD-NA < HCs						
	right Cerebelum_9		1350	3	−51	−45	−4.9

BA, Brodmann’s area; MNI, Montreal Neurological Institute; PD-A, Parkinson’s disease with apathy; PD-NA, Parkinson’s disease without apathy; HCs, healthy controls; NA, Not applicable; - no significant clusters detected.

In contrast, in the slow-4 frequency band, the fALFF value was decreased in the right middle frontal gyrus (*t* = −4.1) but increased in the left cuneus (*t* = 4.2) in patients with PD-A compared to HCs (Figure 2 and Table 3). However, no regions were significantly different between the PD-A and PD-NA groups and also between the PD-NA and HCs.

In the slow-5 frequency band, there was decreased fALFF in the right insula (*t* = −4.7), left insula (*t* = −5.1), left anterior cingulate gyri (*t* = −3.4), left superior frontal gyrus, medial (*t* = −4.1) and left supplementary motor area (*t* = −5.0), along with increased fALFF in the left superior temporal gyrus (*t* = 4.5) and left middle occipital gyrus (*t* = 5.7) in patients with PD-A compared to HCs (Figure 2 and Table 3). However, no regions exhibited significant differences between the PD-A and PD-NA groups. Compared to HCs, patients with PD-NA showed significantly decreased fALFF value in the right cerebellum_9 (*t* = −4.9) (Figure 2 and Table 3).

### Group differences in percent amplitude of fluctuation map

**FIGURE 3:**
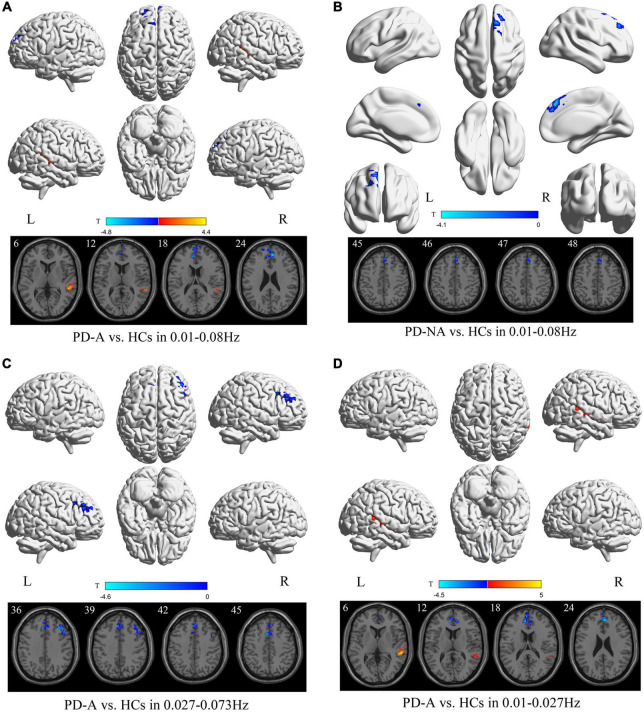
Percent amplitude of fluctuation (PerAF) maps differences among the groups (PD-A vs. HCs and PD-NA vs. HCs) in three frequency bands. The color bar on the bottom indicates the statistical *t* value. The warm (cold) color indicates a significantly increased (decreased) PerAF in the comparison.

In the conventional frequency band, compared to the HCs, patients with PD-A showed significantly increased PerAF value in the right middle temporal gyrus (*t* = 4.4) but decreased in the right anterior cingulate gyri (*t* = −4.8) (Figure 3 and Table 4). Additionally, no regions were significantly different between the PD-A and PD-NA groups. The PerAF value in the right superior frontal gyrus, medial (*t* = −4.1) were decreased in patients with PD-NA compared to HCs (Figure 3 and Table 4).

**TABLE 4:** Differences of percent amplitude of fluctuation (PerAF) values in three frequency bands among the groups.

Contrast	Brain regions	BA	Volume (mm^3^)	MNI coordinates			*t*-value
				X	Y	Z	
**1. Conventional frequency band**							
PD-A vs. HCs	PD-A > HCs						
	right middle temporal gyrus	22	2565	51	−36	6	4.4
	PD-A < HCs						
	right anterior cingulate gyri	32	8208	6	45	24	−4.8
PD-A vs. PD-NA	NA						
PD-NA vs. HCs	PD-NA < HCs						
	right superior frontal gyrus, medial	8	2160	3	36	45	−4.1
**2. Slow-4 band**							
PD-A vs. HCs	PD-A < HCs						
	left supplementary motor area	32	4995	−6	15	45	−4.6
	right middle frontal gyrus	46	2781	33	30	36	−4.3
PD-A vs. PD-NA	NA						
PD-NA vs. HCs	NA						
**3. Slow-5 band**							
PD-A vs. HCs	PD-A > HCs						
	right middle temporal gyrus	22	3375	57	−33	6	5.0
	PD-A < HCs						
	right anterior cingulate gyri	32	4806	3	45	24	−4.5
PD-A vs. PD-NA	NA						
PD-NA vs. HCs	NA						

BA, Brodmann’s area; MNI, Montreal Neurological Institute; PD-A, Parkinson’s disease with apathy; PD-NA, Parkinson’s disease without apathy; HCs, healthy controls; NA, Not applicable; - no significant clusters detected.

In the slow-4 frequency band, compared to HCs, patients with PD-A exhibited decreased PerAF value in the left supplementary motor area (*t* = −4.6) and right middle frontal gyrus (*t* = −4.3) (Figure 3 and Table 4). However, none of the regions varied significantly between the PD-A and PD-NA groups. Furthermore, there was no difference in the slow-4 frequency band between the PD-NA and HCs.

In the slow-5 frequency band, patients with PD-A exhibited decreased PerAF value in the right anterior cingulate gyri (*t* = −4.5) while increased in the right middle temporal gyrus (*t* = 5.0) compared to HCs (Figure 3 and Table 4). However, no regions were significantly different between the PD-A and PD-NA groups and also between the PD-NA and HCs.

### Group differences in regional homogeneity Map

**FIGURE 4:**
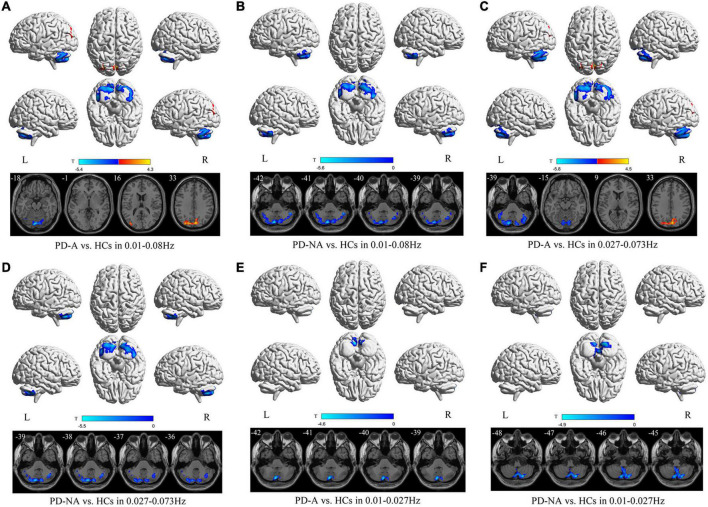
Regional homogeneity (ReHo) maps differences among the groups (PD-A vs. HCs and PD-NA vs. HCs) in three frequency bands. The color bar on the bottom indicates the statistical *t* value. The warm (cold) color indicates a significantly increased (decreased) ReHo in the comparison.

A decreased ReHo value in the left cerebellum_Crus2 (*t* = −5.4) and an increased value in the right cuneus (*t* = 4.3) were observed in patients with PD-A compared to those in the HCs in the conventional frequency band (Figure 4 and Table 5). No regions showed significant variation between the PD-A and PD-NA groups. However, patients with PD-NA had decreased ReHo values in the left cerebellum_Crus2 (*t* = −5.6) compared with the HCs.

**TABLE 5:** Differences of regional homogeneity (ReHo) values in three frequency bands among the groups.

Contrast	Brain regions	BA	Volume (mm^3^)	MNI coordinates			*t*-value
				X	Y	Z	
**1. Conventional frequency band**							
PD-A vs. HCs	PD-A < HCs						
	left Cerebelum_Crus2		27162	0	−81	−18	−5.4
	PD-A > HCs						
	right cuneus	18	7533	6	−81	33	4.3
PD-A vs. PD-NA	NA						
PD-NA vs. HC	PD-NA < HCs						
	left Cerebelum_Crus2		23031	−9	−78	−42	−5.6
**2. Slow-4 band**							
	PD-A < HCs						
	left Cerebelum_Crus2		36126	−12	−81	−39	−5.8
	PD-A > HCs						
	right cuneus	18	7668	6	−81	33	4.5
PD-A vs. PD-NA	NA						
PD-NA vs. HCs	PD-NA < HCs						
	left Cerebelum_Crus2		22464	−12	−81	−39	−5.5
**3. Slow-5 band**							
PD-A vs. HCs	PD-A < HCs						
	left Cerebelum_7b		5940	−6	−78	−42	−4.6
PD-A vs. PD-NA	NA						
PD-NA vs. HCs	PD-NA < HCs						
	right Cerebelum_8		8856	18	−75	−48	−4.9

BA, Brodmann’s area; MNI, Montreal Neurological Institute; PD-A, Parkinson’s disease with apathy; PD-NA, Parkinson’s disease without apathy; HCs, healthy controls; NA, Not applicable; - no significant clusters detected.

Additionally, in the slow-4 frequency band, patients with PD-A showed decreased ReHo values in the left cerebellum_Crus2 (*t* = −5.8), with increased values in the right cuneus (*t* = 4.5) compared to the HCs (Figure 4 and Table 5). However, no regions differed significantly between the PD-A and PD-NA groups. Furthermore, lower ReHo values in the left cerebellum_Crus2 (*t* = −5.5) compared to the HCs (Figure 4 and Table 5).

In the slow-5 frequency band, there was decreased value in the left cerebellum_7b (*t* = −4.6) in patients with PD-A compared to HCs (Figure 4 and Table 5). The PD-A and PD-NA groups did not differ significantly in any region. Patients with PD-NA had significantly lower in the right cerebellum_8 (*t* = −4.9) than those in the HCs (Figure 4 and Table 5).

### Group differences in degree centrality map

**FIGURE 5:**
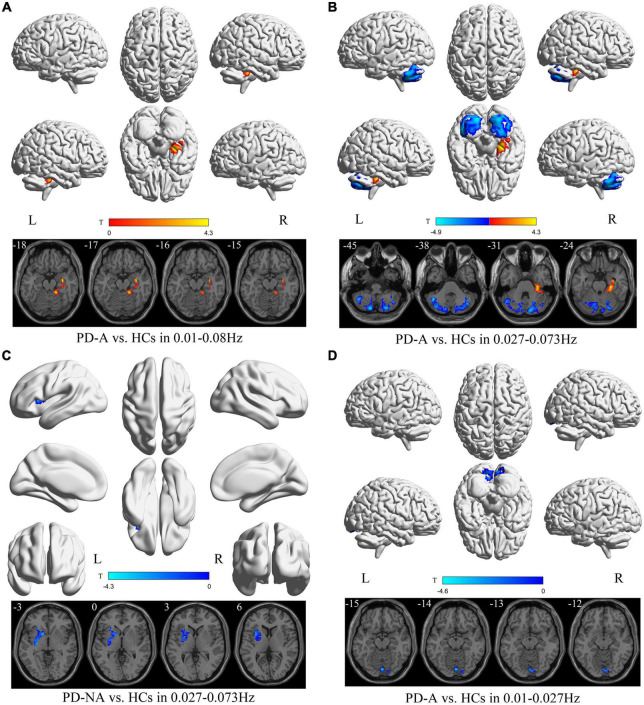
Degree centrality (DC) maps differences among the groups (PD-A vs. HCs and PD-NA vs. HCs) in three frequency bands. The color bar on the bottom indicates the statistical *t* value. The warm (cold) color indicates a significantly increased (decreased) DC in the comparison.

Patients with PD-A had higher DC value in the right hippocampus (*t* = 4.3) in the conventional frequency band than the HCs (Figure 5 and Table 6). However, no regions varied significantly between the PD-A and PD-NA groups. Furthermore, there was no difference in the conventional frequency band between the PD-NA and HCs.

**TABLE 6:** Differences of degree centrality (DC) values in three frequency bands among the groups.

Contrast	Brain regions	BA	Volume (mm^3^)	MNI coordinates			*t*-value
				X	Y	Z	
**1. Conventional frequency band**							
PD-A vs. HCs	PD-A > HCs						
	right hippocampus	20	5427	33	−9	−18	4.3
PD-A vs. PD-NA	NA						
PD-NA vs. HCs	NA						
**2. Slow-4 band**							
PD-A vs. HCs	PD-A < HCs						
	left Cerebelum_7b		36018	−15	−78	−45	−4.9
	PD-A > HCs						
	right parahippocampus	30	5724	27	−24	−24	4.3
PD-A vs. PD-NA	NA						
PD-NA vs. HCs	PD-NA < HCs						
	left pallidum		7452	−12	9	−3	−4.3
**3. Slow-5 band**							
PD-A vs. HCs	PD-A < HCs						
	left Cerebelum_Crus2		2997	0	−84	−15	−4.6
PD-A vs. PD-NA	NA						
PD-NA vs. HCs	NA						

BA, Brodmann’s area; MNI, Montreal Neurological Institute; PD-A, Parkinson’s disease with apathy; PD-NA, Parkinson’s disease without apathy; HCs, healthy controls; NA, Not applicable; – no significant clusters detected.

The DC value decreased in the left cerebellum_7b (*t* = −4.9) and increased in the right parahippocampus (*t* = 4.3) in patients with PD-A compared with the HCs in the slow-4 frequency band (Figure 5 and Table 6). Moreover, no regions differed significantly between the PD-A and PD-NA groups. Compared with the HCs, patients with PD-NA showed reduced DC value in the left pallidum (*t* = −4.3) (Figure 5 and Table 6).

Conversely, in the slow-5 frequency band, patients with PD-A had lower DC values in the left cerebellum_Crus2 (*t* = −4.6) than in the HCs (Figure 5 and Table 6). Additionally, no regions differed significantly between the PD-A and PD-NA groups and also between the PD-NA and HCs.

## Discussion

To our knowledge, this is the first study to systematically evaluate altered ALFF, fALFF, PerAF, ReHo, and DC values in conventional, slow-4, and slow-5 frequency bands separately in patients with PD-A, those with PD-NA, and HCs. We found that the ALFF value in the slow-5 band and fALFF value in the conventional band were more efficient and reliable in differentiating PD-A from PD-NA than in other frequency bands. Furthermore, the ReHo values of cerebellum in the three frequency bands could distinguish PD from HCs. Moreover, abnormal DC value in the hippocampus and parahippocampus was observed separately in the conventional band and in the slow-4 band between PD-A and HCs. Multiple methods can help us to understand the pathological mechanism of Parkinson’s disease from different perspectives.

### Intrinsic brain activity differences between Parkinson’s disease patients and healthy controls

Patients with PD-A and those with PD-NA showed abnormal ALFF, fALFF, PerAF value in the left supplementary motor area, the middle frontal gyrus, and temporo-occipital regions, which included the middle temporal gyrus, superior temporal gyrus, and middle occipital gyrus, compared to the HCs. The decreased activities in the left supplementary motor area and the middle frontal gyrus may be connected with the decline of exercise ability and mood disorders in PD patients, due to its participation in language processing, digital cognition, and so on (Cona and Semenza, 2017). In addition, the temporo-occipital regions are complex domains of the brain, which are assumed to be involved in numerous superior neurological functions, such as self-processing (Blanke and Arzy, 2005), working memory (Deprez et al., 2013), the calculation (Zarnhofer et al., 2012), and reading (Mandonnet et al., 2009) and so on. Previous studies reported that patients with PD had significantly higher ALFF and DC in the temporal gyrus (Guo et al., 2020; Wang et al., 2020), which was consistent with our results. Single-photon emission computerized tomography (SPECT) study also proposed that the temporo-occipital regions participate in the abnormal regional cerebral blood flow in PD (Oishi et al., 2005). Furthermore, we speculate that the left supplementary motor area, the middle frontal gyrus, and temporo-occipital regions are essential in patients with PD. Moreover, we observed that patients with PD showed abnormal ReHo in the cerebellum, relative to the HCs. Previous rs-fMRI studies (Wu and Hallett, 2013; Zeng et al., 2017) reported that abnormalities in the cerebellum were observed in patients with PD, which conformed to our results. Furthermore, we surmise that the decrease ReHo value of cerebellum might be due to the pathological effects. We found that patients with PD-A showed abnormal DC value in the hippocampus and parahippocampus, relative to the HCs. The hippocampus and parahippocampus have long been regarded as critical to memories in space and time (Eichenbaum and Lipton, 2008; Clark and Squire, 2013). The increased DC value in the hippocampus and parahippocampus may be compensatory effect for PD patients maintaining better motor and non-motor functions and this speculation need further investigation.

### Intrinsic brain activity differences between Parkinson’s disease patients with apathy and Parkinson’s disease patients without apathy

Our study found that patients with PD-A had changes in ALFF and fALFF located in the anterior cingulate gyri and middle frontal gyrus, compared with the PD-NA groups. The anterior cingulate gyri play a crucial role in emotional self-control, error recognition, and adaptive responses to fluctuating conditions; therefore, its dysfunction can lead to apathy (Thobois et al., 2017). Moreover, apathy is linked with the frontal cortex, which contributes to working memory and executive functions (Alzahrani et al., 2016). Previous studies have proved that frontal-subcortical circuitry provides a basis for understanding apathy (Bonelli and Cummings, 2007). Our study also reported a similar reduction in fALFF in the middle frontal gyrus in patients with PD-A compared to those with PD-NA, consistent with the declined ALFF signals in this area (Skidmore et al., 2013). A previous rs-fMRI study showed that functional connectivity was decreased between the left planum polare and the right precentral-postcentral gyrus in apathetic frontotemporal dementia (FTD) and patients with PD compared to HCs and non-apathetic patients (Alfano et al., 2021), which revealed that the left planum polare might be involved in developing apathy. However, these results were inconsistent with our findings. We deduced plausible reasons, such as the different disease patients, educational and MMSE levels, and the confounding influence (for example, ON or OFF medication) on the results. Combined with these findings, we believe that the anterior cingulate gyri and middle frontal gyrus are the major hubs of PD-A.

### Intrinsic brain activity differences between the frequency bands

Additionally, our study observed that the abnormal ALFF, fALFF in some brain regions were varied in specific frequency bands in patients with PD-A compared with the HCs and those with PD-NA. Specifically, patients with PD-A had abnormal fALFF values in the bilateral insula in slow-5 band compared to HCs, fALFF value in the right middle frontal gyrus in conventional band compared to PD-NA. PD-A showed dysfunctions of ALFF in the middle occipital gyrus limited to the slow-5 band compared with that in the HCs. The slow-5 band (0.010–0.027 Hz) is linked with the incorporation of large-scale neural networks and long-distance connectivity and is localized primarily to the occipital gyrus and insula, among other (Zuo et al., 2010; Baria et al., 2011). Moreover, the anterior cingulate gyri also showed frequency-dependent in slow-5 band for the PD-A and PD-NA comparison. Therefore, our results implied that insula, the middle frontal gyrus, the middle occipital gyrus, and the anterior cingulate gyri may be involved in apathetic behavior, which may associate with frequency specificity.

In our studies, we found a very interesting phenomenon that there are very rare “pure apathy” PD patients in clinical treatment. Patients with apathy often combined with cognitive impairment, depression, and so on (Pagonabarraga et al., 2015; Mele et al., 2020). Although we make use of setting exclusion criteria and using statistical comparisons to removed confounding factors, however those combined factors should not be ignored in future studies.

### Limitations

The current study has some limitations. First, although the depression, anxiety, and cognitive impairment were not significantly different between PD-A and PD-NA groups, these confounding factors still can’t be ignored. Second, we assessed the severity of apathy in PD-A patients by the AS, which is an international standard scale for assessing apathy, while multiple comprehensive tools should be used for the assessment and classification of apathy, and multiple neuroimaging modalities should be used to explore the diagnosis for apathy in future studies. Third, due to the relatively small sample size, we did not perform the correlation analysis between regional rs-fMRI parameters and clinical measures of the disease. The correlation analysis in large-sample and follow-up studies should be evaluated in the future.

## Conclusion

Our study showed the differences in ALFF, fALFF, PerAF, ReHo, and DC value in different frequency bands among patients with PD-A, those with PD-NA, and the HCs. Therefore, the ALFF value in the slow-5 band and fALFF in conventional band are potential effective local parameters in distinguishing PD-A from PD-NA. Finally, our findings provide new insights for further investigating frequency-dependent resting-state functional disruption in patients with PD-A.

## Data availability statement

The raw data supporting the conclusions of this article will be made available by the authors, without undue reservation.

## Ethics statement

The studies involving human participants were reviewed and approved by China-Japan Union Hospital of Jilin University Ethics Committee. The patients/participants provided their written informed consent to participate in this study.

## Author contributions

HX wrote the manuscript. HX, MZ, LL, and YC conceived of the idea and performed the literature review. MZ and ZW performed the data analysis. HX, YC, and YY contributed to the data collection. All authors interpreted the results, reviewed the manuscript, and joined the discussion of the manuscript.

## Footnotes

1
http://www.fil.ion.ucl.ac.uk/spm/software/spm12
